# Behavioral intention to have risky sex in young men and women: The role of sexual excitation and assertiveness

**DOI:** 10.1371/journal.pone.0232889

**Published:** 2020-05-21

**Authors:** Reina Granados, Nieves Moyano, Juan Carlos Sierra

**Affiliations:** 1 Nursing Department, Faculty of Health Sciences, University of Granada, Granada, Spain; 2 Department of Evolutionary Psychology and Education, Faculty of Humanities and Sciences Education, University of Jaén, Jaén, Spain; 3 Mind, Brain and Behavior Research Center (CIMCYC), University of Granada, Granada, Spain; Universita degli studi di Padova (Padua University), ITALY

## Abstract

Due to the complex role of sexual excitation in risky sexual behaviors, this study aimed to disentangle this phenomenon by jointly analyzing the combined role of three forms of sexual excitation: genital and subjective, and individual´s propensity. Therefore, we examined the relationship between the components of the Dual Control Model, that is, propensity for sexual excitation/inhibition, in addition to genital and subjective arousal, and sexual assertiveness and intention to engage in casual sexual encounters in which sexual risk was implicitly or explicitly present. The sample consisted of 99 heterosexual young adults (55 men and 45 women) with ages ranging from 18 to 32 years. Participants performed an experiment in the laboratory, which involved them watching a sexual clip and then being presented with two erotic excerpts (stories) depicting casual sexual encounters in which there was an existence of implicit and explicit sexual risks. In men, the propensity for sexual inhibition was the most determining variable in preventing them from sexual risk-taking. In women, intention to engage in risky sexual behaviors was better determined by their propensity for sexual excitation and sexual assertiveness in negotiating the use of contraceptive methods. This research highlights the relevance of excitation and inhibition as a trait, in addition to subjective arousal and sexual assertiveness in intention to engage in risky sexual behaviors.

## Introduction

The Dual Control Model of sexual response (DCM) proposes that the sexual excitation and inhibition systems present in each individual are essential for adequate sexual functioning, which is made possible by the balance and interaction between the two ☯1]. These two systems are relatively independent, and individuals differ in their propensity for sexual excitation and sexual inhibition [1–3]. Thus, it has been observed that high levels of sexual inhibition are associated with greater vulnerability to sexual dysfunctions, particularly when high sexual inhibition is paired with low levels of sexual excitation [2]. In contrast, low levels of sexual inhibition are associated with a greater likelihood to engage in risky sexual behaviors, particularly when paired with high sexual excitation [4]. In this context, inhibition of the sexual response is considered a relevant adaptive mechanism for sexual risk-taking [5]. Individuals generally have a base level of inhibition that prevents a sexual response from taking place until the situation or sexual stimulus has been assessed as non-threatening [1–2,6]. As proposed by the DCM, in certain individuals, this adaptive mechanism might be absent, or the propensity for inhibition might be lower, implying a higher probability of getting involved in risky sexual situations [3,5]. If these individuals additionally show high propensity for excitation, a sexual response may develop even in presence of a threat [2], thus associating this scenario with risky sexual behaviors [3,5]. In general terms, although the presence of sexual excitation does imply lower risk control, it is sexual inhibition that effectively counteracts this effect [6].

Sexual activity provides individuals with positive reinforcements, but it also implies a cost in terms of risk [1,7]. In this regard, risky sexual behaviors (RSBs) increase the probability of experiencing negative consequences as a result of sexual activity [8], potentially affecting individuals’ physical, mental, and social well-being [9]. Some examples of RSBs are the use of alcohol and/or drugs in sexual encounters [10–11], non-use of contraceptive methods, casual sex, and sex with multiple partners [12, 13]. Some of the physical consequences of these behaviors are sexually transmitted infections (STIs) [7,14], and unplanned pregnancies [15]. More than one million individuals, worldwide, contract STIs every day, and unplanned pregnancies are also common. This leads to significant effects on health and quality of life [14, 16]. Definitions of sexual risk behaviors also include both psychological and social well-being. Therefore, some of their negative consequences are feelings of fear [17,18], guilt [18], regret [19], and stigma [17, 20]. Despite our knowledge about RSBs and their negative consequences, prevalence among youngsters is still high [21]. This may be explained by their low perception of risk [22]. Therefore, the lack of concordance between knowledge and attitudes toward health and taking risks in sexual interactions is shown [22,23]. Other authors attribute risky behaviors to lack of profound knowledge about (de)protection and its negative consequences [24], near-fatalistic expectations for the future [25], lack of sexual health education and the need to strengthen social support networks [26], lack of social/communicative abilities, such as sexual assertiveness [27], higher levels of sexual sensation seeking [28,29], and also sexual decision-making [30], among others.

Due to the consequences and implications of RSBs in several sexual health aspects, some studies have addressed the factors associated with some indicators of RSBs. A research line that derived from the DCM, which was mostly based on a cross-sectional methodology, has explored the role that sexual excitation/inhibition plays in the performance of risky behaviors by both men and women. Janssen et al. [31] demonstrated that, in heterosexual men, the propensity for sexual excitation (SES) positively predicted the number of sexual partners in the past year, whereas inhibition, due to the threat of performance consequences (SIS2), negatively predicted the number of sexual partners with whom no condoms were used in the past three years. Similar results were later obtained by Bancroft et al. [32] and Peterson et al. [33], who additionally demonstrated that sexual inhibition, due to the threat of performance failure (SIS1), positively predicted the number of lifetime occasional sexual partners and sexual encounters without a condom in the past year. In homosexual men, SES and SIS1 positively predicted the number of casual sexual partners, whereas SIS2 negatively predicted it [4]. In addition, SIS2 negatively predicted the frequency of unprotected anal and oral sex [4,34]. SES was higher in the high sexual risk group in both homosexual and heterosexual men [7].

The relationship between the dimensions of the DCM and RSBs has also been explored in women using the Sexual Excitation/Sexual Inhibition Inventory for Women (SESII-W) [35], and the Sexual Excitation/Sexual Inhibition Inventory for Women and Men (SESII-W/M) [36]. The number of sexual partners and sexual risk-taking have been found to be positively predicted by SE and negatively predicted by SI [37–39]. Likewise, higher levels of SE have been associated with inconsistent or nonexistent condom use, along with engaging in sexual contact under the influence of alcohol or drugs [40]. SIS2 has also been negatively associated with intention to engage in sexual contact with men who have had more than ten sexual partners with no condom use [41].

One of the variables that have been associated with RSBs is sexual assertiveness [27], that is, the ability of individuals to initiate sexual contact, reject undesired sexual contact, and negotiate the use of contraceptives [42]. This has been related to the number of sexual partners [43] and condom use [44–45]. Although the relationship between sexual assertiveness and sexual excitation has been reported in previous research [46], the study of their joint role in sexual risk-taking has not been thoroughly addressed.

Traditional gender roles have also been associated with RSBs. Indeed, men tend to show more active sexual behaviors (i.e., courtship, take the initiative for a sexual encounter, dominant, etc.), while women tend to perform a more passive role (i.e., sensitive, romantic or submissive, etc.) [47,48]. Moreover, men often report more RSBs than women [47–49].

Sexual arousal has been conceptualized as a complex phenomenon that involves multiple response systems with physiological, psychological (cognitive and affective) and behavioral components [e.g., 2,3,50,51–53]. In his review, Janssen [54] defined sexual arousal as “an emotional/motivational state that can be triggered by internal and external stimuli and that can be inferred from central (including verbal), peripheral (including genital), and behavioral (including action tendencies and motor preparation) responses” (p. 710). Subjective sexual arousal is better defined by cognitive processes as an individual´s experience or feeling, which is related to the affective and cognitive evaluation of sexual excitation [55]. Genital sexual arousal is the most frequent sexual response associated with it [56]. According to Chivers et al. [57], the most common way of measuring sexual arousal has been through self-reported measures (e.g., items answered by Likert-type scales, scales, inventories or mobile lever), and sexual arousal, specifically, has been measured by phallometry, vaginometry and thermography. There is debate about which measure is more appropriate to better register sexual arousal, as notable variation in female sexual concordance between these measures has been evidenced. Therefore, it is relevant to measure sexual arousal based on all these three forms: as a trait, genital and subjective.

The present laboratory study was conducted in order to gain further insight into the relationship between the components of the DCM and RSBs. The study had the following objectives: (a) to explore behavioral intention to engage in sexual contact in two contexts: one with implicit sexual risk and one with explicit sexual risk; (b) to analyze the relationship between the sexual inhibition/excitation patterns proposed by the DCM and the arousal experienced in a specific situation–genitally and subjectively- with the behavioral intention to engage in sexual contact; and (c) to analyze the role of sexual assertiveness regarding behavioral intention to engage in sex in both contexts.

The following hypotheses were developed:

H1. A higher percentage of participants will decide to initiate a sexual encounter in a context with implicit sexual risk than in a context with explicit sexual risk. According to Becoña [58], decision-making processes take the consequences produced by an act into account, this act being rejected when it is assessed as disadvantaged.
H2. According to previous research [12,37], greater propensity for sexual excitation has been associated with a greater number of sexual partners and casual encounters. We consider that, in both contexts, participants with higher levels of sexual excitation -propensity, genital and/or subjective- will report a higher behavioral intention to engage in sexual contact.
H3. In both contexts, participants with higher levels of sexual inhibition will report a lower behavioral intention to engage in sexual contact [31,37].
H4. In both contexts, individuals with higher assertiveness to initiate sexual contact will report a higher behavioral intention to engage in such contact; in contrast, individuals with higher assertiveness to refuse unwanted sexual contacts and greater ability to negotiate the use of contraceptive methods will show a lower behavioral intention to engage in sexual behaviors [27].
H5. Finally, gender differences will be observed. Therefore, in both contexts, men will show a higher intention to initiate a sexual encounter than women. This hypothesis is based on the traditional gender roles that still persist in our society, in which men are allowed to play a more sexually active role than women [59].

Previous research into the relationship between the DCM components and sexual assertiveness, and also into their potential joint influence on RSBs, is lacking. Therefore, the following hypotheses were tested to examine their possible interactions:

H6. Higher SES and higher assertiveness to initiate sexual contact have been related to higher RSBs [12,27]. Therefore, a positive relationship between them is expected.
H7. SIS has been negatively related to RSBs [4,12]. According to the DCM, SIS acts as a protective factor against this type of behavior [1–2,31,60]. Moreover, assertiveness to refuse unwanted sexual contacts and assertiveness to negotiate the use of contraceptive methods have been negatively related to RSBs [27]. Thus, a positive relationship between SIS and both dimensions of assertiveness is expected.

## Materials and methods

### Participants

The sample consisted of 99 heterosexual young adults from southern Spain (54 men, 45 women). Ages ranged from 18 to 30 years (*M* = 20.93, *SD* = 2.42) in men, and from 19 to 32 years (*M* = 21.43, *SD* = 3.18) in women. The age of first intercourse ranged from 15 to 20 years (men: *M* = 16.98, *SD* = 1.41; women: *M* = 16.17, *SD* = 1.62). All participants reported having had previous sexual intercourse. At the time of the study, 9.3% of the men and 21.4% of the women were in a relationship. The mean number of sexual partners was 5.60 (*SD* = 5.84) in men and 6.83 (*SD* = 9.33) in women.

The inclusion criteria were being aged between 18 and 35 years-old and having a heterosexual orientation. The exclusion criteria were having a psychological disorder, a sexual or a medical condition, and using medication (e.g., antidepressants, antihypertensives, antipsychotics), and/or drugs or alcohol that might interfere with sexual function.

### Measures

#### Demographic and sexual history questionnaire

This questionnaire includes questions about age, education level and sexual orientation, measured by the Heterosexual–Homosexual Rating Scale [61], relationship status (0 = Not involved in a steady relationship, 1 = In a steady relationship), age of first sexual intercourse, and number of sexual partners. Questions were also raised about psychological, medical, or sexual problems, whether the participants were receiving some type of treatment (medical and/or psychological), and the consumption of drugs and alcohol.

#### Sexual excitation and sexual inhibition

In men, the Spanish version of the Sexual Inhibition/Sexual Excitation Scales (SIS/SES) [31] by Granados et al. [62] was used to determine propensity for sexual inhibition/excitation. The SIS/SES consist of 34 items distributed into four scales: Sexual Excitation Scale (SES), Sexual Inhibition Scale 1 or Inhibition due to the threat of performance failure (SIS1), Sexual Inhibition Scale 2 or Inhibition due to the threat of risk of being caught while having sex (SIS2), and Sexual Inhibition Scale 3 or Inhibition due to the threat of performance consequences (SIS3). Higher scores indicate greater sexual excitation/inhibition. Reliability for the Spanish version, indicated by Cronbach´s alpha values, was .87 for SES, .83 for SIS1, .68 for SIS2, and .49 for SIS3. In women, sexual excitation and sexual inhibition were assessed with the Spanish version of the SESII-W [35,37], which comprises 33 items distributed into eight subfactors–four grouped into SE and four grouped into SI. The reliability coefficients in previous Spanish samples have been adequate [37]. The Spanish version has adequate values of reliability for each component: .84 for SE and .76 for SI.

#### Sexual assertiveness

Sexual assertiveness was assessed with the Sexual Assertiveness Scale (SAS) [42,63]. This scale comprises 18 items grouped into three dimensions: Initiation (α = .85), Refusal (α = .76), and Pregnancy/Sexually Transmitted Disease (STD) prevention (α = .85).

#### Subjective sexual arousal

Subjective sexual arousal was evaluated with the Spanish versions [64] of the Ratings of Sexual Arousal (RSA) and the Ratings of Genital Sensations (RGS), both included in the Multiple Indicators of Subjective Sexual Arousal [55]. The RSA estimates subjective sexual arousal using 5 items that are answered on a 7-point Likert scale (from 1 = no sexual arousal at all–to 7 = extremely sexually aroused). The RGS measures the level of genital sensations with an 11-item checklist scale (from 1 = no genital sensation–to 11 = multiple orgasm). The RSA showed an internal consistency reliability of .90, and its correlation with the RGS was .73 [63].

#### Genital sexual arousal

The genital response of men was measured with an indium-gallium strain gauge [65,66]. This device measures the changes in penile circumference when an erection is taking place. Vaginal photoplethysmography [67] was used to measure the genital response of women. This device measures vaginal pulse amplitude (VPA) [50,68]. The Biopac MP 150 system was used, with AcqKnowledge software for data acquisition and processing (BIOPAC Systems, Inc., Goleta, CA, USA). Each VPA signal was visually inspected and movement artefacts were removed. After this, peak-to-peak amplitudes were calculated. The genital sexual responses were standardized within participants to *z*-scores. Genital responses were defined in terms of differences between sexual and baseline stimuli.

#### Stimulus materials

As a baseline measure, two neutral content films (geographic documentaries) were used. Each one lasted for 3 minutes. Participants watched one of these neutral films before each sexual clip. Two sexual clips were used in the study, lasting for 3 minutes each and showing heterosexual couples having oral and vaginal sex [69–73]. After watching the sexual clip, participants were presented with one of two erotic excerpts (stories), describing a potential sexual encounter between the participant and an attractive partner. The stories were written in the second person so as to involve the participant in them, and their narrative evolved from a casual encounter to an imminent sexual encounter, similarly to those previously used in other research studies [74,75]. The *explicit sexual risk context* included a sexual risk situation in the form of lack of contraceptive methods whereas the *implicit sexual risk context* did not refer to the presence or absence of these methods. These sexual risk contexts were derived from previous research [see 50,74]; that is, casual sexual intercourse with no specific reference to any type of preventive method.

#### Behavioral intention to engage in sexual contact

In order to measure intention to engage in casual sexual contact, once participants had read the erotic excerpts (i.e., explicit sexually risky and implicit sexually risky contexts), they were asked to answer the following question: “In the situation that you have just read about, how would you behave? Please select only one option.” Response options were: 1) “I would take the initiative in order to have intercourse” (Initiate sex); 2) “I would wait for the other person to take the initiative and accept having intercourse” (Wait); and 3) “I would not continue with the sexual contact so as to avoid having intercourse” (Not continue).

### Procedure

#### Pre-experimental instructions and initial procedures

The sample was obtained by convenience sampling. Participants were recruited using flyers, noticeboards and advertisements in social networks (i.e., Facebook). Individuals, who were willing to take part, were first required to complete a questionnaire to better determine if they met the inclusion criteria. Before arriving at the laboratory, volunteers were informed by e-mail and by telephone of the experimental procedure, the stimuli, the devices to be used, the purpose of the study, and what their participation consisted of. Eligible participants received study information by e-mail along with a copy of informed consent. Women were not evaluated during menstruation. In addition, they were asked to abstain from caffeine, alcohol, and sexual activity during the 24 hours prior to the experimental session to minimize possible physiological sources that might vary the responses [76]. Participants signed the informed consent in the laboratory. All volunteers were undergraduate students at the time the study was conducted.

#### Arrival at laboratory

Once at the laboratory, participants were shown the photoplethysmographs and were trained in their placement. They were also given the informed consent document to read and sign. Afterward, they answered the SIS/SES or SESII-W, and the SAS on a computer. The experimental sequence was carried out in a soundproof room under the same temperature, light and humidity conditions in all cases. After providing the explanation, the researcher left the room, and once alone, the participant fitted the photoplethysmograph. With the photoplethysmograph in place, the participant sat comfortably in front of a screen and remained on hold for a 5-minute adaptation period before the experiment began.

#### Sexual arousal induction and sexual-risk context

All participants were presented with two experimental sequences: (a) *implicit sexual risk context* (viewing the neutral and sexual films plus reading the implicit sexual risk erotic story) and (b) the *explicit sexual risk context* (viewing the neutral and sexual films plus reading the explicit sexually risky erotic story). These sequences were counterbalanced in order to control any possible effects of the order of presentation of the stimuli. At the end of each sequence, participants answered the subjective measures of sexual arousal (RSA and RGS) and selected one option depending on their intention to engage in sexual contact–initiate sexual contact, wait or not continue-. All instructions were given over the screen. Approximate participation time was 60 minutes. This study was approved by the Ethics Committee on Human Research of the University of Granada.

### Data analysis

1) First, we conducted zero-order correlations among the evaluated variables. 2) In order to check sexual arousal induction after the sexual film, we calculated the difference in genital arousal between neutral and sexual visual stimulus through non-parametric tests for related samples using Wilcoxon´s test. 3) An assessment was made of participants’ behavioral intention by sexual risk context to which they had been exposed -implicit and explicit-. 4) A multivariate analysis of variance (MANOVA) was performed to determine whether there were differences in intention to engage in sex–take the initiative, wait or not continue-, for both implicit and explicit sexual contexts in the following variables: sexual excitation–propensity for SES/SIS, genital and subjective arousal- and sexual assertiveness. These variables were considered independent variables and behavioral intention was considered the dependent variable, which was coded as: take the initiative = 1, wait = 2, and not continue = 3. Wilks’ lambda (λ) was used to determine the existence of statistically significant differences in all dependent variables. Post-hoc comparisons were computed with the Bonferroni test. The partial eta squared (η2) statistic was used to estimate effect size. 5) Following the procedure recommended by Pedhazur [77], hierarchical multiple regression analyses were conducted to evaluate the predictive role of propensity for sexual excitation and inhibition, and of sexual assertiveness, in behavioral intention to engage in casual sexual contact in both implicit and explicit sexual risk contexts. The mediating effect of subjective and genital sexual arousal was explored. The following regression models were tested in men and women in both contexts (i.e., implicit and explicit sexual risks) to determine the direct and mediating effects of the variables assessed:

- Model 1 explored the relationship between 1) SES, SIS1, SIS2, and SIS3 in men, SE and SI in women, and the three dimensions of sexual assertiveness (i.e., Initiation, Refusal, and Pregnancy/STD prevention), and 2) behavioral intention (i.e., take the initiative, wait, or not continue). The sexual excitation/inhibition variables were introduced in Block 1, and the three dimensions of sexual assertiveness were introduced in Block 2.
- Model 2 explored the relationship between sexual excitation/inhibition and sexual assertiveness on both genital and subjective sexual arousal (RSA and RGS).
- Model 3 explored the relationship between 1) both genital and subjective sexual arousal (RSA and RGS) and 2) behavioral intention.
- Model 4 analyzed the mediating effect of genital and subjective sexual arousal (RSA and RGS) in the relationship between propensity for sexual excitation/inhibition and sexual assertiveness (independent variables), and intention to engage in casual sexual contact (dependent variable).

All statistical analyses were performed by SPSS v.22.

## Results

Zero-order correlations among the evaluated variables can be seen in Tables 1 and 2 for men and women, respectively. For the implicit sexual risk context, behavioral intention to have sex was unrelated to any of the sexual-related variables in men, while in women, greater intention to initiate sex was related to both propensity for SES and subjective arousal. For the explicit sexual risk context, SIS2 and SAS-P/STD were positively correlated with more secure sexual behavior in men, while in women, more propensity for SE correlated with greater behavioral intention to have sex, while SI and SAS-P/STD correlated with more secure behavior. Surprisingly, more genital arousal was also related to more secure behavior.

**Table 1:** Zero-order correlations among the evaluated variables in men.

	1	2	3	4	5	6	7	8	9	10	11	12	13	14
**1. Relationship status**		.14	.01	-.09	-.11	.22	.18	-.13	.01	.02	.18	-.03	-.03	.04
**2. Age of first sexual intercourse**	.14	_	-.20	.19	.02	.20	.21	.00	.14	.16	.12	-.33*	-.04	.27
**3. Number of sexual partners**	.01	-.20	_	-.14	.07	-.26	-.17	-.07	.13	-.09	-.12	.04	-.24	-.28
**4. Behavioral intention to have sex**	-.13	-.01	-.19	_	-.07	.22	.13	.01	-.06	-.25	-.09	-.04	.08	.13
**5. SES**	-.11	.02	.07	-.11	_	-.24	-.26	-.17	.16	.35*	.38**	.01	-.31*	-.24
**6. SIS1**	.22	.20	-.26	.06	-.24	_	.28*	.28*	.13	-.01	.10	-.06	.09	.13
**7. SIS2**	.18	.21	-.17	.39**	-.26	.28*	_	.44**	-.21	-.12	-.06	-.19	.39**	.44**
**8. SIS3**	-.13	.00	-.07	.26	-17	.28*	.44**	_	.01	.11	.15	-.23	.16	-.29*
**9. Genital arousal**	.03	.01	.03	-.08	.13	.03	-.06	.09	_	.39**	.25	-.09	-.20	-.02
**10. Subjective arousal–RSA**	-.03	.12	-.12	-.02	.38**	-.10	-.06	.15	.23	_	.85***	-.24	-.11	-.02
**11. Subjective arousal–RGS**	.09	.28	-.28	.00	.42**	-.08	.06	.06	.36**	.84***	_	-.12	-.02	.07
**12. SAS-Initiation**	-.03	-.33*	.04	-.18	.01	-.06	-.19	-.23	-.18	-.12	-.16	_	-.00	-.14
**13. SAS-Refusal**	-.03	-.04	-.24	.10	-.31*	.09	.39**	.16	-.14	-.02	-.06	-.00	_	.28*
**14. SAS-P/STD**	.04	.27	-.28	.41**	-.02	.13	.44**	.29*	.05	.07	.18	-.14	.28*	_

Above the diagonal: Implicit sexual risk context. Under the diagonal: Explicit sexual risk context. Behavioral intention to have sex = 1 = Initiate, 2 = Wait, 3 = Not continue. SES = Sexual Excitation Scale. SIS1 = Sexual Inhibition Scale 1 (Inhibition due to the threat of performance failure). SIS2 = Sexual Inhibition Scale 2 (Inhibition due to negative consequences such as the threat of risk of being caught while having sex). SIS3 = Sexual Inhibition Scale 3 (Inhibition due to the threat of performance consequences). RSA = Ratings of Sexual Arousal; RGS = Ratings of Genital Sensations; Physiological arousal indicated by penile circumference. SAS-Initiation = Initiation assertiveness. SAS-Refusal = Refusal assertiveness. SAS-P/STD = Pregnancy/STD prevention assertiveness.

*p < .05

**p < .01

***p < .001.

**Table 2:** Zero-order correlations among the evaluated variables in women.

	1	2	3	4	5	6	7	8	9	10	11	12
**1.**		_	_	-.03	,05	-.10	-.04	.15	.23	-.19	-.36*	.00
**2. Age of first sexual intercourse**	_	_	-.30	-.22	-.31	.25	-.10	.21	.41*	-.01	.20	.38*
**3. Number of sexual partners**	_	-.30	_	-.27	.58***	-.05	-.12	.33	.25	-.00	-.46**	-.56**
**4. Behavioral intention to have sex**	-.26	.18	-.32	_	-.35*	.10	.00	-.46**	-.49**	-.27	.04	.10
**5. SE**	.05	-.31	.58**	-.37*	_	-.32*	-.08	.59***	.33*	.30*	-.31*	-.16
**6. SI**	-.10	.25	-.05	.35*	-.32*	_	.06	.01	-.89	-.25	.08	.20
**7. Genital arousal**	-.08	-.14	-.12	.32*	-.01	.12	_	-.02	-.02	-11	-.03	.16
**8. Subjective arousal—RSA**	.06	.16	.27	-.23	.57***	.01	.01	_	.83***	.17	-.23	-.09
**9. Subjective arousal—RGS**	.15	.42*	.10	-.13	.40**	-.02	-.16	.86***	_	.16	-.09	-.08
**10. SAS-Initiation**	-.19	-.01	-.00	-.23	.30*	-.25	-.06	.10	.06	_	.18	-.03
**11. SAS-Refusal**	-.36*	.19	-.46**	.22	-.31*	.08	-.02	-.27	-.27	.18	_	.27
**12. SAS-P/STD**	.00	.38*	-.56**	.44**	-.16	.20	.24	-.01	.03	-.03	.27	

Above the diagonal: Implicit sexual risk context. Under the diagonal: Explicit sexual risk context. Behavioral intention to have sex = 1 = Initiate, 2 = Wait, 3 = Not continue. SE = Sexual Excitation. SI = Sexual Inhibition. RSA = Ratings of Sexual Arousal. RGS = Ratings of Genital Sensations. Genital arousal indicated by vaginal pulse amplitude. SAS-Initiation = Initiation assertiveness. SAS-Refusal = Refusal assertiveness. SAS-P/STD = Pregnancy/STD prevention assertiveness.

*p < .05

**p < .01

***p < .001.

Differences in genital arousal between neutral and sexual clips were examined in both experimental sequences by gender. In men, significant differences were found in physiological arousal between the neutral and sexual clips (neutral visual stimulus 1 –sexual visual stimulus 1 (Z = -6.36, *p* < .001) and neutral visual stimulus 2 –sexual visual stimulus 2 (Z = -6.00, *p* < .001), with higher genital arousal during the sexual clips (*M*_*neutral clip1*_ *=* 101.19, *SD* = 17.60; *M*_*sexual clip1*_ *=* 117.30, *SD* = 20.33; *M*_*neutral clip2*_ *=* 101.25, *SD* = 14.00; *M*_*sexual clip2*_ *=* 116.90, *SD* = 20.32). In women, significant differences were also found in genital arousal between the neutral and sexual clips (neutral visual stimulus 1 –sexual visual stimulus 1 (Z = -5.60, *p* < .001) and neutral visual stimulus 2 –sexual visual stimulus 2 (Z = -5.11, *p* < .001) with higher genital arousal during the sexual visual stimulus (*M*_*neutral clip1*_ *=* 0.05, *SD* = 0.07; *M*_*sexual clip1*_ *=* 0.10, *SD* = 0.01; *M*_*neutral clip2*_ *=* 0.06, *SD* = 0.01; *M*_*sexual clip2*_ *=* 0.10, *SD* = 0.01).

Next, we explored the distribution of men and women as a function of their behavioral intention to engage in sexual contact depending on the context (implicit or explicit sexual risk). Table 3 lists the percentages for each behavioral intention option. In both contexts, the highest percentages corresponded to subjects who would decide to take the initiative, with men scoring higher in the implicit sexual risk context (75.5%). The percentage of individuals who reported to “wait” was similarly distributed for both contexts and by gender. Finally, the percentage of participants who would not continue the sexual contact was higher in the sexually explicit risk context than in the implicit sexual risk context, although more women than men chose this option (33.3%). In spite of these differences, the distribution of percentages between the implicit and the explicit context was significant for both men (Z = -3.42, *p* < .001) and women (Z = -3.62, *p* < .001). According to H1, most of the participants chose the option to initiate the sexual encounter in the implicit sexual risk context, although these differences were significant for women. In addition, and regarding the hypothesis about gender differences, although men, apparently, in contrast to women, reported greater intention to engage in sex, gender differences were found for the explicit (Z = -2.15, *p* < .05) but not for the implicit context (Z = −1.62, *p* = .104) (H5). Thus, men, in the explicit context are more willing to have sex. In contrast, women are more conservative in this context.

**Table 3:** Distribution of men and women as a function of their behavioral intention to have sex depending on the context (implicit or explicit sexual risk).

	Men		Women	
Behavioral intention	Implicit sexual risk	Explicit sexual risk	Implicit sexual risk	Explicit sexual risk
	*n* (%)	*n* (%)	*n* (%)	*n* (%)
**Initiate sex**	40 (75.5)	25 (47.2)	29 (64.4)	17 (37.8)
**Wait**	12 (22.6)	19 (35.8)	14 (31.1)	13 (28.9)
**Not continue**	1 (1.9)	9 (17)	2 (4.4)	15 (33.3)

### Differences in sexual excitation–propensity, genital and subjective- and assertiveness as a function of behavioral intention

In the implicit sexual risk context, when participants were asked about their behavioral intention to engage in sex, only one man and two women reported to not continue. Consequently, we excluded these cases, in order to assure more equally distributed groups if there were differences in the examined variables based on their behavioral intention. Therefore, we found no significant differences for any of the examined variables between those who reported to initiate sex or to wait. In the explicit sexual risk context, the men who would take the initiative, in contrast to those who reported to wait, were those with low levels of SIS2 (*F*_(2,47)_ = 7.18, *p* = .002). In addition, men with higher refusal assertiveness more often reported to wait in contrast to initiate sex, and men with high Pregnancy/STD prevention assertiveness (*F*_(2,47)_ = 5.22, *p* = .009) were more likely to report to wait or to not continue. See Table 4.

**Table 4:** MANOVA of implicit and explicit sexual risk contexts in men.

	Implicit sexual risk context									Explicit sexual risk context								
	Initiate sex (*n* = 40)		Wait (*n* = 12)					Initiate sex (*n* = 25)		Wait (*n* = 19)		Not continue (*n* = 9)				
	*M*	*SD*	*M*	*SD*	*F*	*p*	*n*^*2*^	*M*	*SD*	*M*	*SD*	*M*	*SD*	*F*	*p*	*n*^*2*^
**SES**	42.51	6.44	41.33	7.68	0.36	.701	.015	42.88	6.10	41.82	6.33	40.71	9.59	0.32	.731	.014
**SIS1**	20.26	4.89	21.67	3.98	0.39	.679	.017	20.50	4.54	20.94	4.84	20	6.92	0.09	.911	.004
**SIS2**	9.25	2.49	9.83	2.33	1.34	.271	.055	**8.25**_**b**_	**2.19**	**10.76**_**a**_	**2.31**	**10.70**	**1.70**	**7.29**	**.001**	**.250**
**SIS3**	10.17	2.05	9.75	1.82	1.13	.333	.047	9.50	2.13	10.88	1.76	10.71	1.38	2.93	.064	.064
**Genital arousal (range = -1.32 to 59.13)**	16.68	10.49	15.01	10.53	1.37	.248	.029	16.38	16.60	16.95	12.42	12.32	9.19	0.47	.631	.020
**Subjective arousal—RSA**	18.11	5.72	16.42	5.79	0.78	.381	.017	17.99	5.24	19.94	6.88	14.42	7.59	1.99	.149	.081
**Subjective arousal—RGS**	3.29	1.41	3.33	1.50	0.01	.921	.000	3.37	1.31	3.76	1.79	2.86	1.95	0.85	.436	.436
**SAS-Initiation**	**12.31**	**3.87**	**10.08**	**4.81**	**5.76**	**.006**	**.200**	12.54	3.68	11.82	4.26	11.29	7.57	0.253	.778	.011
**SAS-Refusal**	11.48	5.15	13.58	6.13	0.65	.526	.028	**10.33**_**b**_	**4.73**	**14.94**_**a**_	**4.85**	**10.72**	**7.20**	**4.83**	**.013**	**.180**
**SAS-P/STD**	14.80	6.19	15.17	5.49	0.71	.499	.030	**12.46**_**b**_	**5.79**	**17.35**_**a**_	**5.11**	**19.14**_**a**_	**5.05**	**6.27**	**.004**	**.222**

SES = Sexual Excitation Scale. SIS1 = Sexual Inhibition Scale 1 (Inhibition due to the threat of performance failure). SIS2 = Sexual Inhibition Scale 2 (Inhibition due to negative consequences such as the threat of risk of being caught while having sex). SIS3 = Sexual Inhibition Scale 3 (Inhibition due to the threat of performance consequences). Genital arousal: indicated by penile circumference. RSA = Ratings of Sexual Arousal; RGS = Ratings of Genital Sensations. SAS-Initiation = Initiation assertiveness. SAS-Refusal = Refusal assertiveness. SAS-P/STD = Pregnancy/STD prevention assertiveness. Significant results in bold type. Different subscripts indicate significant differences in the pair comparison.

As regards women, for the implicit sexual risk context, those with higher levels of SE (*F*_(2,39)_ = 5.67, *p* = .022), RSA (*F*_(2,39)_ = 11.75, *p* = .001) and RGS (*F*_(2,39)_ = 13.94, *p* = .001) were more numerous at reporting that they would take the initiative in comparison to those who decided to wait. In the explicit sexual risk context, women with higher levels of SE (*F*_(2,39)_ = 6.57, *p* = .003) and initiation assertiveness (*F*_(2,39)_ = 4.13, *p* = .024) tended to report that they would take the initiative, while those with higher levels of SI (*F*_(2,39)_ = 6.20, *p* = .005), reported to wait or to not continue. Women who reported greater Pregnancy/STD prevention assertiveness (*F*_(2,39)_ = 3.41, *p* = .043) tended to report that they would not continue with the sexual contact or would wait instead of initiating sex (see Table 5).

**Table 5:** MANOVA of implicit and explicit sexual risk contexts in women.

	Implicit sexual risk context							Explicit sexual risky context								
	Initiate sex (*n* = 29)		Wait (*n* = 14)					Initiate sex (*n* = 17)		Wait (*n* = 13)		Not continue (*n* = 15)				
	*M*	*SD*	*M*	*SD*	*F*	*p*	*n*^*2*^	*M*	*SD*	*M*	*SD*	*M*	*SD*	*F*	*p*	*n*^*2*^
**SE**	**51.04**	**7.00**	**45.46**	**5.01**	**5.67**	**.022**	**.122**	**53.31**_**a**_	**6.90**	**45.08**_**b**_	**5.22**	**47.62**	**6.51**	**6.57**	**.003**	**.252**
**SI**	39.78	5.63	42.00	6.15	0.55	.461	.013	**37.06**_**b**_	**4.91**	**41.92**_**a**_	**3.93**	**43.38**_**a**_	**6.27**	**6.20**	**.005**	**.241**
**Genital arousal (range = .01 to .40)**	0.10	0.09	0.10	0.08	0.03	.863	.001	0.06	0.05	0.08	0.07	0.12	0.11	1.83	.174	.086
**Subjective arousal—RSA**	**26.61**	**6.32**	**19.15**	**6.45**	**11.75**	**.001**	**.236**	21.88	6.11	18.01	8.35	18.38	6.51	1.11	.338	.054
**Subjective arousal—RGS**	**4.22**	**1.37**	**2.62**	**1.04**	**13.94**	**.001**	**.268**	3.56	1.15	3.23	1.92	3.08	1.61	0.37	.693	.019
**SAS-Initiation**	14.30	4.72	12.84	4.04	0.09	.348	,023	**15.88**_**a**_	**4.18**	**11.38**_**b**_	**5.04**	**12.92**	**3.57**	**4.13**	**.024**	**.175**
**SAS-Refusal**	18.85	3.68	19.62	3.95	0.36	.552	.009	18.94	4.04	17.84	3.93	20.31	3.68	1.30	.284	.063
**SAS-P/STD**	17.56	6.09	19.46	3.67	1.07	.306	.028	**16.06**_**b**_	**6.69**	**17.46**	**4.94**	**21.15**_**a**_	**3.39**	**3.41**	**.043**	**.149**

SE = Sexual Excitation. SI = Sexual Inhibition. Genital arousal indicated by vaginal pulse amplitude. RSA = Ratings of Sexual Arousal. RGS = Ratings of Genital Sensations. SAS-Initiation = Initiation assertiveness. SAS-Refusal = Refusal assertiveness. SAS-P/STD = Pregnancy/STD prevention assertiveness. Significant results in bold type. Different subscripts indicate significant differences in the pair comparison.

### Predictive variables of behavioral intention in men

In the implicit sexual risk context, none of the sexual excitation/inhibition and sexual assertiveness dimensions significantly predicted behavioral intention to have sex (Model 1). Next, Model 2 was tested. In this model, only SES was found to significantly predict RSA (β = .36, *p* = .015) and explained 11% of its variance (adjusted *R*^*2*^ = .11, *p* < .011) (*F*_(1, 50)_ = 6.99, *p* = .011); in other words, subjects with a higher propensity for sexual excitation reported higher subjective sexual arousal (RSA) when viewing the sexual clip. In Model 3, no significant correlations were found between sexual arousal, both genital and subjective, and behavioral intention to have sex. Due to the lack of significance of the variables tested in the prediction of behavioral intention, Model 4 was not run. Therefore, in men and in an implicit sexual risk context, none of the predictive factors tested were able to predict behavioral intention.

**Fig 1:**
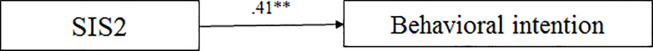
Path model for the prediction of behavioral intention in the explicit context for men. **p < .01.

In the explicit sexual risk context, Model 1 showed a significant correlation between SIS2 and behavioral intention (β = .41, *p* = .003), explaining 17% of the variance (adjusted *R*^*2*^ = .17, *p* < .001) of behavioral intention to not engage in casual sexual contact (*F*_(1,52)_ = 11.34, *p* = .001). Model 2 revealed that only SES has a significant influence on subjective sexual arousal, both on RSA (β = .39, *p* = .008), explaining 12.7% of the variance (adjusted *R*^*2*^ = .12, *p* = .038) (*F*_(4,45)_ = 2.77, *p* = .038), and on RGS (*β* = .48, *p* = .001), explaining 15.3% of the variance (adjusted *R*^*2*^ = .15, *p* = .023) (*F*_(1,49)_ = 10.96, *p* = .002). Model 3, which tested the predictive power of genital and subjective sexual arousal (RSA and RGS) over behavioral intention to engage in casual sexual contact, did not show any significant correlations. Model 4 was not tested for this reason. Thus, in men, in a context in which sexual risk was explicit, behavioral intention to not engage in sex was only predicted by sexual inhibition due to negative consequences (SIS2) (see Fig 1).

### Predictive variables of behavioral intention in women

**Fig 2:**
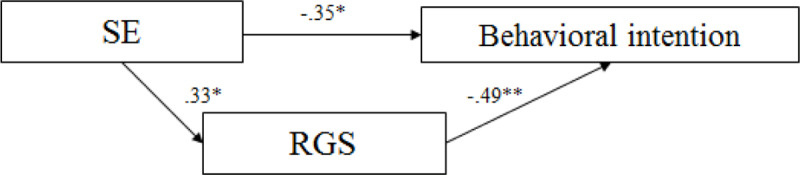
Path model for the prediction of behavioral intention in the implicit context for women. *p < .05, **p < .01.

In the implicit sexual risk context, Model 1 indicated that SE predicted behavioral intention (β = -.35, *p* = .018), explaining 10.3% of the variance (adjusted *R*^*2*^ = .10, *p* < .05) of intention to initiate sex (*F*_(1,44)_ = 6.07, *p* = .018). In Model 2, SE predicted RGS (β = .33, *p* = .030), explaining 9% of its variance (adjusted *R*^*2*^ = .09, *p* < .05) (*F*_(1,41)_ = 5.05, *p* = .030). Model 3 indicated an effect of RGS on behavioral intention to engage in casual sexual contact (β = -.49, *p* = .001), explaining 23% of the variance (adjusted *R*^*2*^ = .23, *p* < .001) (*F*_(1,41)_ = 13.22, *p* = .001); no significant correlations were found with RSA or genital response. Finally, when Model 4 was tested to determine if SE and RGS jointly influenced behavioral intention, SE ceased to be significant (*β* = -.23, *p* = .11) while RGS remained significant (β = -.42, *p* = .005). This result evidenced the mediating effect of subjective sexual arousal (RGS), which is influenced by the individual´s propensity for sexual excitation (SE). In this model, RGS explained 26% of the variance (adjusted *R*^*2*^ = .26, *p* < .001) of behavioral intention to engage in casual sexual contact (*F*_(1,41)_ = 8.23, *p* = .001). Together, in a context where sexual risk is implicit, women with greater propensity for sexual excitation and who subjectively experienced greater arousal would be more likely to initiate sex (see Fig 2).

**Fig 3:**
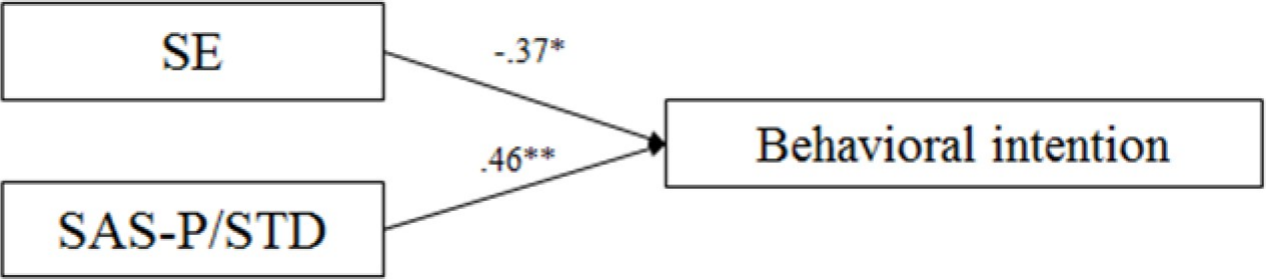
Path model for the prediction of behavioral intention in the explicit context for women. *p < .05, **p < .01.

Finally, in the explicit sexual risk context, SE (β = -.37, *p* = .013) and SAS-P/STD prevention assertiveness (β = .46, *p* = .005) were found to be significantly correlated with behavioral intention to engage in casual sexual contact, explaining 25% of its variance (adjusted *R*^*2*^ = .25, *p* < .001) (*F*_(1,44)_ = 8.32, *p* = .001) (Model 1). While greater SE predicted a higher probability to engage in sex, more prevention assertiveness better predicted not continuing the sexual encounter. Model 2 revealed that SE had an effect on RSA (*β* = .61, *p* = .000) and on RGS (β = .46, *p* = .005). The model predicted 36% of the variance in RSA (adjusted *R*^*2*^ = .359, *p* = .000) (*F*_(1,44)_ = 25.63, *p* = .000) and 14.4% of the variance in RGS (adjusted *R*^*2*^ = .144, *p* = .008; *F*_(1,41)_ = 7.89, *p* = .008). In Model 3, neither RSA nor RGS were associated with behavioral intention to engage in casual sexual contact, thus ruling out their mediating effect. Due to the lack of significance of the mediating variables in behavioral intention, Model 4 was not tested. In short, the best predictors of women´s behavioral intention, in a context with explicit sexual risk, were their propensity for sexual excitation and Pregnancy/STD prevention assertiveness, with no mediation of either their genital or subjective arousal. In particular, greater propensity for excitation would foster their intention to have sex, while their greater assertiveness related to the risk of Pregnancy/STD would make them avoid having sex (see Fig 3).

## Discussion

The aim of this study was to analyze the role of the components of DCM, along with sexual assertiveness, in intention to engage in casual sexual contact in both implicit and explicit risk contexts. Our findings indicate that a greater percentage of both men and women would take the initiative to continue a sexual contact in an implicit sexual risk context as opposed to an explicit sexual risk context. This was to be expected, as these decisions are taken depending on the assessment of their positive or negative consequences, rejecting any actions that might be disadvantageous [58]. In particular, in men, only propensity for sexual inhibition plays a significant role in intention not to have sex in an explicit risk context. However, propensity for sexual excitation, genital response, subjective arousal or sexual assertiveness have little to do with their behavioral intention to have sex, contrary to our hypotheses. For women, their propensity for sexual excitation and their subjective arousal better predict their likelihood to initiate sex in an implicit risk context, while their propensity for sexual excitation and their assertion to negotiate contraceptive methods are associated with their intention to not initiate risky sex in an explicit risk context, which partially supports our hypotheses.

The DCM indicates that the inhibition system of sexual response acts as an adaptive mechanism with regard to sexual risks, preventing a sexual response from taking place until the situation has been assessed as non-threatening [1–3,5]. It is worth noting that almost 85% of the men and 66.7% of the women in the study reported intention to engage in sexual intercourse in an explicit risk context, either by taking the initiative or by letting their sexual partner take that step. This may be due to a low perception of risk [78–79] as a result of either failing to identify the consequences or considering them to be remote or not related to oneself [58,80]. According to the DCM, every individual has a base level of sexual inhibition [1] that allows for a sexual response to take place once the situation or sexual stimulus has been evaluated as non-threatening [2]. Paradoxically, it may also occur that a real risk is not assessed as such due to factors such as obtaining immediate pleasure, the long time interval between the risky act and its consequences, the assurance that medical advances will solve any problem, or the cultural justification of risky behaviors as correct [81–82]. This study also confirms the hypothesis that men, compared to women, are keener to take the initiative to engage in casual sexual contact. This might be related to the presence of traditional gender roles characterized by the belief that men exhibit active sexual behaviors (i.e., seduction, decisiveness, initiative), whereas women show passive traits (i.e., sensitivity, romanticism, submission) [59].

Regarding the variables associated with behavioral intention to engage in sex, we observed that, for men, in the implicit sexual risk context, there are no variables associated with behavioral intention to have sex or not. However, in the context in which risk is explicitly present, men with higher propensity for sexual inhibition, in particular related to the threat of being caught while having sex (SIS2), are more likely to refuse to have sex. Also, men who report less assertiveness to refuse sex more frequently report to wait, while men who report to be less able to negotiate the use of contraceptive methods with their partners are more likely to decide to wait or to not continue having sex. Therefore, assertiveness of both types is associated with a lower intention to engage in risky sexual contacts. However, when the predictive model was tested, the only predictor factor that emerged as crucial to predict sex refusal was SIS2. These findings are consistent with the DCM in that, due to the variability in individual propensity for sexual inhibition, certain individuals have low or no inhibition, and therefore show a higher probability of engaging in risky sexual contact [1–2,6]. Based on previous research, individuals with lower SIS2 also tend to show a lower propensity for sexual sensation-seeking or erotophilia, which are both associated with RSBs [83–84]. On the other hand, although sexual assertiveness is associated with behavioral intention in men, these variables do not seem crucial as predictors. Refusing sexual assertiveness has not been explored much in men [27] but it has been widely studied in women, in whom its deficit has been associated with a higher number of sexual partners [43]. Additionally, in men, Pregnancy/STD prevention assertiveness has been negatively associated with the number of unprotected sexual contacts [85] and positively associated with protected sex [44,85] and consistent condom use [85].

Regarding women, those who indicate higher propensity for excitation and higher subjective sexual arousal, when they are presented with sexual stimuli, also report higher behavioral intention to engage in sexual contact in an implicit sexual risk context, confirming the proposed hypothesis that postulates these variables as predictors. By not perceiving an explicit sexual risk, these women might be evaluating the situation as non-threatening and thus the basal sexual inhibition threshold may be surpassed by sexual excitation, facilitating the decision and subsequent sexual response [1–3,5].

In the context in which sexual risk is explicit, women with higher propensity for sexual excitation report greater intention to take the initiative to engage in sexual contact. Moreover, in line with the hypotheses proposed in this study, women who are more prone to sexual inhibition and women with greater assertiveness related to the negotiation of contraceptive methods are more likely to say “no” to sex in the presence of a sexual risk concerning this issue. In previous studies, both sexual inhibition [38–39] and Pregnancy/STD prevention assertiveness [43–44] have proven to be protective variables with regard to sexual risk-taking. It was also observed that, even though the women who reported an intention not to continue the sexual contact were physiologically aroused, they reported an intention not to engage in the RSB. This corroborates the protective role of SI and Pregnancy/STD assertiveness. Several studies in women have shown the relationship between these two variables and RSBs. In fact, a higher score in SE has been associated with sexual risk-taking [38], a higher risk of contracting an STI [40], a higher number of sexual partners [37,39], younger age of first sexual intercourse [37], and a higher frequency of unprotected sexual contact [39], among others. Initiation assertiveness has also been associated with a higher number of sexual partners [43] and lower condom use [86].

According to our findings, propensity for sexual excitation/inhibition as a trait, and subjective sexual arousal as a state, were more relevant for risky sexual behavior than genital response. At this point, we should consider that the sample of participants was physiologically aroused. Therefore, it is likely that, in line with their intention to engage in sex, all participants were, at a certain level of arousal or in “the heat of the moment”, considering the control and artificial setting of the laboratory. Therefore, genital arousal probably played some role in fostering their intention to engage in risky sex.

In short, certain studies have associated RSBs with the measures derived from the DCM [38], and others have found a relationship between this type of behavior, and subjective and physiological measures of sexual arousal [75]. However, to our knowledge, this is the first study that combines propensity for sexual excitation and inhibition (DCM), genital and subjective sexual arousal, and sexual assertiveness in the decision-making process on sexual risk-taking in men and women. Another novelty that can be observed in this study is that the included variables related to sexual risk-taking behave differently according to the sex of the participants. Regarding our findings, the most relevant variable in sexual risk-taking in men is propensity for sexual inhibition, specifically inhibition related to the threat of being caught having sex (SIS2). For this reason, more research should be conducted on SIS2 as a variable that may inhibit RSBs. Moreover, although this study did not show any significant findings regarding SIS3 (i.e., fear of the consequences of sexual contact), further research is also recommended on this topic, as this was the factor that showed to have the closest relationship with RSBs. In women, SE and Pregnancy/STD prevention assertiveness are elements that should be taken into account in explaining intention to engage in RSBs, which also justifies conducting further research on these topics. These variables should be considered when designing RSB prevention and intervention programs.

Certain limitations of this study should be mentioned. The results cannot be extrapolated to the general population because, although this is common practice in psychophysiological studies on human sexuality [67], the sampling method was not random. Additionally, the sample only comprised heterosexual young adults. For this reason, more diverse population groups should be recruited (i.e., non-heterosexual, adolescents, elderly and clinical populations, etc.). Furthermore, although the reliability value for SIS3 was low, in this work its use was considered necessary. This weak reliability value could be explained by the heterogeneity of the content of items that comprised this subfactor (i.e., consequences of risky sexual behavior and the presence of pain felt by oneself or by the sexual partner during sex). Although the reliability value was inadequate, we decided to use this factor because its items represented threatening situations where sexual inhibition would act adaptively and protectively [1]. This factor could also provide valuable information that would contribute to preventing sexual risk behavior and pain in excitation situations.

## Conclusions

In summary, the present study highlights the role that sexual excitation/inhibition, sexual arousal and sexual assertiveness play in intention to engage in risky sexual behaviors. We also emphasize that these factors differ between men and women. Therefore, propensity for sexual inhibition is the most determining variable for intention of sexual risk-taking in men, while propensity for sexual excitation and sexual assertiveness in negotiating the use of contraceptive methods are the variables that are more strongly associated with intention of sexual risk-taking in women. Based on our findings, different approaches should be taken in order to address prevention for sexual risk behaviors by gender. Considering that sexual excitation increases sexual risk-taking and sexual inhibition mostly prevents these behaviors, more studies should be conducted to go much further into the role that these variables play in RSBs. Furthermore, we emphasize the need to measure propensity for sexual excitation/inhibition, as it would help sexual health professionals to prevent and intervene in order to improve the balance between both systems. Taken together, a balance between both systems would provide healthier sexual behavior. To strike this balance, individuals should be more aware of the risks and consequences of their behaviors. In this way, and as explained by the theoretical framework of the DCM, sexual inhibition would act as a preventive system when the evaluation of the sexual situation were labeled as threatening. Sexual education programs are needed to make people aware of their sexual health and their rights to develop a risk-free sex life. Education programs should have a stronger impact on the development of sexual assertiveness and abilities to negotiate condom use as they are crucial to consent to desirable and healthy sexual encounters. Taken together, we should care about the prevention and reduction of the negative consequences derived from this type of sexual behaviors such as STIs, unplanned pregnancies and feelings of fear and guilt, among others. Finally, although previous research has associated RSBs with certain measures of the DCM [39], and others authors have related these behaviors to subjective and genital measures of sexual arousal [71], as far as we know, this is the first study to combine the analysis of both propensity for sexual excitation/inhibition, subjective and genital sexual arousal, and sexual assertiveness in risky sexual decision-making in two different sexual contexts in both genders.
